# Integration of Virtual and Augmented Reality Into Obstetric Nursing and Midwifery Education: Systematic Review

**DOI:** 10.2196/90252

**Published:** 2026-10-05

**Authors:** Mariana Luisa Firmiano, Ana Paula Assunção Moreira, Flaviana Vely Mendonça Vieira, Carla Sa-Couto, Alexandrina Cardoso

**Affiliations:** 1 School of Medicine and Biomedical Sciences, University of Porto, Rua de Jorge Viterbo Ferreira, 228, Porto, 4050-313, Portugal, 351 931352400; 2 Nursing School, Federal University of São Paulo, São Paulo, São Paulo, Brazil; 3 Faculty of Nursing, Federal University of Goiás, Goiânia, Goiás, Brazil; 4 RISE-Health, Faculty of Medicine, University of Porto, Porto, Portugal; 5 RISE-Health, Nursing School of University of Porto, University of Porto, Porto, Portugal

**Keywords:** midwifery, virtual reality, augmented reality, obstetric nursing, nursing education, nurse midwives, simulation-based learning

## Abstract

**Background:**

Technologies such as virtual reality (VR) and augmented reality (AR) have been increasingly incorporated into nursing education to support the development of cognitive, psychomotor, and behavioral competencies. In midwifery, immersive environments offer opportunities to simulate high-risk or low-frequency clinical scenarios, strengthening students’ confidence and preparedness for professional practice. However, there is still significant variability in how VR and AR are pedagogically implemented, with limited understanding of their theoretical grounding, instructional design, and educational outcomes.

**Objective:**

This systematic review aimed to synthesize and critically evaluate the characteristics of educational programs using VR and AR to develop competencies among nursing and midwifery students and professionals.

**Methods:**

This review followed the PRISMA (Preferred Reporting Items for Systematic Reviews and Meta-Analyses) 2020 and Synthesis Without Meta-Analysis (SWiM) guidelines and was registered in PROSPERO (International Prospective Register of Systematic Reviews). A comprehensive search was conducted in August 2024 across PubMed, Web of Science, Scopus, and Cumulative Index to Nursing and Allied Health Literature (CINAHL) databases. Eligible studies were experimental, quasi-experimental, or observational, and involved undergraduate or postgraduate students in nursing, obstetric nursing, or midwifery, as well as certified midwives or nurse-midwives. Two independent reviewers conducted screening, data extraction, and quality appraisal using Joanna Briggs Institute (JBI) tools and the Medical Education Research Study Quality Instrument (MERSQI) scale. Data were synthesized narratively according to predefined thematic categories.

**Results:**

Four studies published between 2021 and 2024 met the inclusion criteria, encompassing 634 participants (360 midwifery students and 274 midwives or nurse-midwives). Two randomized controlled trials and 2 quasi-experimental studies were included. Interventions used VR in 3 studies and AR in one, simulating scenarios such as normal and complicated childbirth, neonatal resuscitation, and emergency obstetric procedures. Most interventions focused on developing technical competencies, while one also addressed teamwork and communication. Only 2 studies explicitly reported theoretical or pedagogical frameworks, and 2 mentioned alignment with the INACSL Healthcare Simulation Standards of Best Practice. Structured briefing or debriefing was rarely described. All studies assessed learning outcomes (Kirkpatrick level 2), and 3 included participants’ satisfaction (level 1). Methodological quality, measured by MERSQI, ranged from 10.0 to 14.5, with a mean score of 12.9 (SD 2.0), indicating moderate to high quality.

**Conclusions:**

Evidence suggests that VR and AR can enhance learning in obstetric nursing and midwifery education by providing safe, experiential, and interactive learning environments. However, the limited use of theoretical frameworks and structured instructional design highlights the need for more pedagogically grounded interventions. Future research should prioritize the integration of learning theories, validated assessment tools, and best-practice simulation standards to strengthen the educational impact and curricular integration of immersive technologies in nursing and midwifery education.

## Introduction

### Background

The integration of virtual reality (VR) and augmented reality (AR) has demonstrated promising effectiveness in health education, particularly in the development of cognitive, psychomotor, and behavioral competencies [1-3]. These technologies enable immersion in interactive 3D environments, allowing for the safe practice of complex procedures and the visualization of anatomical and physiological phenomena that are difficult to observe in real clinical settings [1,4]. When applied in a planned manner and supported by clear educational objectives, they foster clinical reasoning, decision-making [5], and student engagement [6].

Nursing education has explored the potential of these technologies across different clinical areas [2], and obstetric nursing and midwifery, in particular, has benefited from the ability to simulate complex clinical scenarios, contributing to the development of technical and nontechnical competencies essential for safe care of women throughout the perinatal period [7,8].

Despite advances in research, there remains significant variability in how VR and AR are implemented within nursing education programs, reflecting the diversity of pedagogical approaches and levels of curricular integration reported in the literature [5,9,10]. Given the inherent complexity of immersive environments, challenges related to students’ cognitive load may be exacerbated, particularly when the degree of immersiveness and the instructional design are not aligned with the learning objectives [11,12]. Therefore, it is relevant to understand how immersive technologies have been applied in obstetric nursing and midwifery education and to what extent their implementation is grounded in solid pedagogical and theoretical principles [13,14].

In this context, a systematic synthesis of the literature is essential to identify how VR and AR have been integrated into obstetric nursing and midwifery education, evaluating the presence of theoretical foundations, instructional models, and assessment strategies that guide their pedagogical application. This analysis contributes to the development of evidence-based educational practices for the integration of these technologies into nursing and midwifery education.

### Objective

This systematic review aimed to synthesize and critically evaluate the characteristics of educational programs that use VR and AR technologies to develop obstetric competencies among nursing and midwifery students and professionals.

## Methodology

### Study Design

This systematic review was conducted in accordance with the PRISMA (Preferred Reporting Items for Systematic Reviews and Meta-Analyses; Checklist 1) guidelines and the Cochrane Handbook for Systematic Reviews of Interventions [15,16]. The study protocol was developed by the research team and registered in PROSPERO (International Prospective Register of Systematic Reviews; registration number: CRD42025635292). No ethical approval was required.

### Information Sources and Search Strategy

The search was conducted on August 14, 2024, in the following databases: PubMed, Web of Science, Scopus, and Cumulative Index to Nursing and Allied Health Literature (CINAHL).

The search strategy was structured based on the PIO framework (Population, Intervention, Outcome). The population included undergraduate and postgraduate students in nursing, obstetric nursing, or midwifery, as well as practicing midwives and nurse-midwives. The interventions comprised educational programs using VR and AR as pedagogical tools for developing clinical competencies in obstetrics. The outcomes of interest encompassed theoretical and pedagogical models, instructional design, assessment strategies, and competencies addressed by the interventions.

The terms were organized into 3 conceptual blocks and adapted to the syntax of each database. No filters were applied regarding language, publication date, or document type. The complete search strategy is presented in Multimedia Appendix 1.

### Eligibility Criteria

Eligible studies were primary research with experimental, quasi-experimental, or observational designs, published in peer-reviewed journals. The population included undergraduate nursing students, obstetric nursing and midwifery students, postgraduate obstetric nursing and midwifery students, and certified midwives and nurse-midwives.

Interventions were required to involve extended reality (XR) technologies, operationalized in this review as VR or AR applications used for clinical simulation with active learner interaction. VR interventions were eligible when they involved head-mounted or headset-based immersive delivery and first-person interaction within a simulated clinical environment, such as manipulating virtual objects, selecting or performing clinical actions, or responding to scenario-based clinical cues. AR interventions were eligible when they involved digital overlays integrated into the real-world environment, accessed through mobile or wearable devices, and required interaction with virtual elements as part of the simulated clinical activity. Passive 360-degree video, noninteractive video-based learning, desktop-based simulation, standard e-learning, mobile applications without AR overlays or immersive delivery, physical simulations without VR or AR integration, training conducted outside educational contexts, and studies in general nursing without a specific focus on obstetric or immediate neonatal care were excluded (Multimedia Appendix 1).

### Study Selection Process

The search results were imported into Rayyan (Qatar Computing Research Institute Hamad Bin Khalifa University) [17]. After duplicate removal, 2 reviewers (MLF and APM) independently and blindly screened the studies across 3 successive phases: titles, abstracts, and full texts. Interrater reliability was assessed using the final subset of 33 records evaluated at the full-text stage. Agreement was reached for 28 of the 33 decisions (84.8%), yielding a Cohen kappa coefficient of 0.52, which indicates moderate agreement. Each reviewer systematically recorded the reasons for exclusion using a structured format based on the eligibility criteria. Discrepant decisions were initially discussed between the 2 reviewers to reach consensus. When consensus was not possible, the studies were referred to 2 additional reviewers (AC and CSC), who independently reassessed them using the same criteria. Final inclusion or exclusion decisions were made after discussion among the reviewers, considering the predefined methodological criteria and the additional assessments provided.

Most disagreements involved borderline eligibility decisions related to the operational definition of immersive technology and clinical simulation. In particular, discrepancies concerned the distinction between fully immersive VR and 360-degree video or other noninteractive formats, the minimum level of user interaction required for inclusion, and whether the intervention involved scenario-driven simulation within an obstetric or immediate neonatal care context. These issues were resolved by applying the predefined eligibility criteria, which required immersive VR or AR with active learner interaction and a specific focus on obstetric or immediate neonatal care education.

### Data Extraction

Data extraction was conducted independently by 2 reviewers (MLF and APM) using a standardized form, specifically developed for this review based on the study objectives. The form was pilot-tested on 2 included studies, allowing adjustments to ensure clarity and consistency in data collection.

Information was extracted regarding study characteristics, population, and educational intervention. These data included: study title, authors, year of publication, and country; study design; participants’ professional background and educational level; learning theory adopted; instructional design models; the Bloom Revised Taxonomy domains targeted by the intervention [18]; Kirkpatrick levels used for evaluating the interventions [19]; type of clinical environment and simulated context; technical and nontechnical skills addressed; data collection methods and assessment instruments (type, content, validity, and timing); and data analysis methods. Regarding technological features, we extracted system-level characteristics related to immersive delivery, including device type and interaction modality with virtual elements. Additional data included supporting educational materials, use of complementary teaching strategies, duration of the simulation, reported implementation challenges, funding sources, and outcomes related to knowledge, skills, attitudes, and learning experience. Discrepancies were resolved by consensus or by consulting a third reviewer (CSC). The data were organized using Microsoft Excel spreadsheets.

### Risk-of-Bias and Methodological Quality Assessment

Risk of bias was assessed independently by two reviewers (MLF and APM) using the Joanna Briggs Institute (JBI) critical appraisal tools [15], applied according to the methodological design of each included study.

For randomized controlled trials (RCTs), the revised JBI checklist comprising 13 items was used [20]. These are organized into 5 core domains: participant selection and allocation, delivery of the intervention or exposure, assessment, detection and measurement of outcomes, participant retention, and statistical conclusion validity.

For quasi-experimental studies, the revised JBI checklist with 9 items was applied [21], covering the following domains: temporal-procedural bias, selection and allocation bias, confounding bias, bias in the delivery of the intervention or exposure, bias in the assessment, detection and measurement of outcomes, and bias related to participant retention.

Each item in the checklists was classified as “yes,” “no,” “unclear,” or “not applicable,” in accordance with the guidance provided in the respective tools. Disagreements between reviewers were resolved by consensus.

In addition, the Medical Education Research Study Quality Instrument (MERSQI) [22] was used to assess the methodological quality of studies from the perspective of health professions education research. The instrument comprises 6 domains: study design, sampling, type of data, validity of instruments, statistical analysis, and educational outcomes. Each domain has a maximum score of 3 points, with the total score ranging from 5 to 18 points.

Uncertainties and disagreements were addressed and resolved through consensus. Studies were not blinded with regard to authorship, institutional affiliation, or publishing journal.

### Data Synthesis Strategy

In line with the review objectives, a narrative synthesis approach was planned a priori and conducted following the Synthesis Without Meta-Analysis (SWiM) reporting guidelines [23]. A meta-analysis was not performed due to substantial heterogeneity across the included studies, including differences in intervention characteristics (VR vs AR), study designs, outcome measures, assessment instruments, and timing of evaluation. In addition, quantitative data were reported inconsistently, with some studies presenting means and SDs, while others reported percentages or did not provide sufficient data, further precluding meaningful statistical pooling.

The extracted information was organized into predefined thematic categories, including study characteristics, educational interventions, theoretical and pedagogical foundations, technologies used, assessment instruments, and main findings. The results were presented descriptively and analytically, allowing comparisons based on shared methodological elements. Supporting tables and summary charts were developed to facilitate comparative analysis, and quantitative evidence was summarized where appropriate. Findings were synthesized by grouping results according to outcome domains (knowledge, skills, attitudes, and learning experience) and by examining the direction and consistency of results across studies.

## Results

The database search retrieved 4237 records. After the removal of 1330 duplicates, 2907 titles were screened, resulting in 244 records selected for abstract assessment. Of these, 33 full-text articles were evaluated, and 4 studies were included in the review. Figure 1 presents the PRISMA flow diagram of the selection process.

**Figure 1.**
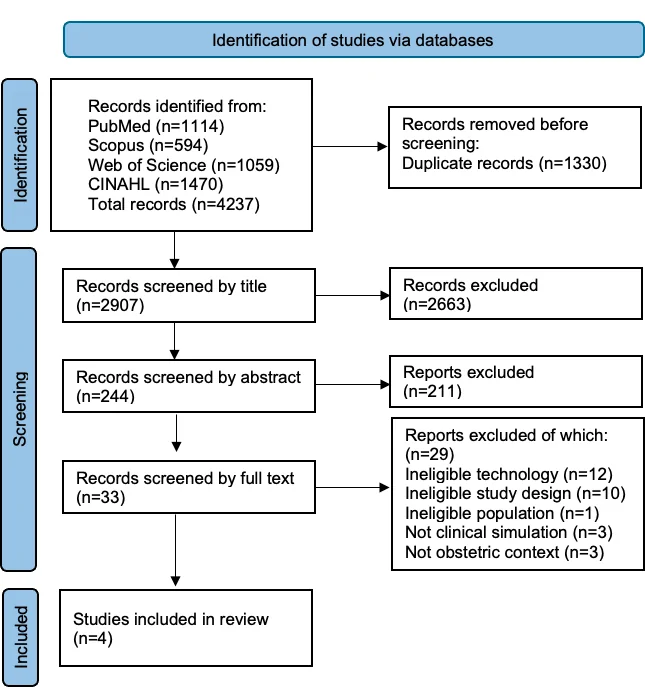
PRISMA (Preferred Reporting Items for Systematic Reviews and Meta-Analyses) flow diagram of the present study.

### Characteristics of the Studies

The 4 included studies [24-27] were published between 2021 and 2024 and represented diverse geographical and educational contexts. Three studies involved undergraduate midwifery students [24-26], while one multicenter study included certified midwives and nurse-midwives working in maternity settings [27]. Overall, the review comprised 634 participants, including 360 midwifery students and 274 practicing midwives or nurse-midwives.

The methodological designs included 2 RCTs: one with 6-month follow-up comparing the use of VR with instructional video or digital guide [27], and another comparing VR with low-fidelity clinical simulation [25]. In addition, one quasi-experimental study with a control group compared VR with traditional teaching methods [26], and another quasi-experimental study without a control group used pre- and postintervention measures to evaluate the impact of AR across different simulated clinical scenarios [24]. Detailed study characteristics are presented in Table 1.

**Table 1.** Study characteristics.

Study (year)	Country	Type of study	Population and sample	Study objectives	XR^a^ Type	Simulation scenario
Vogel et al (2024) [24]	Germany	Quasi-experimental, without a control group, (pre- and posttest design)	Midwifery students, n=133	To investigate whether AR^b^ simulation training influences midwifery students’ subjective perceptions of knowledge, confidence, and practical skills in emergency situations.	AR	Emergency tocolysis, preparing a pregnant woman for cesarian section, neonatal resuscitation
Öner and Turfan (2024) [25]	Türkiye	RCT^c^, (posttest only design)	Midwifery students, n=92	To compare the impact of VR^d^ simulation with low-fidelity simulation in teaching newborn care to obstetric nursing students.	VR	Initial care of the newborn
Zhao et al (2024) [26]	China	Quasi-experimental, with a control group, (posttest only design)	Midwifery students, n=135	To evaluate the effect of case-based learning method with VR simulation technology on midwifery courses.	VR	Eutocia (normal birth), dystocia, umbilical cord prolapse, and neonatal asphyxia and resuscitation
Umoren et al (2021) [27]	Nigeria and Kenya	RCT, 6 months follow-up	Midwives and nurse-midwives, n=274	To assess the impact of VR simulations using electronic Helping Babies Breathe or video for the maintenance of neonatal resuscitation skills in health care workers in resource-scarce settings.	VR	Routine newborn care, initial resuscitation, and prolonged resuscitation with positive pressure ventilation

a XR: extended reality.

b AR: augmented reality.

c RCT: randomized controlled trial.

d VR: virtual reality.

### Description of the Educational Intervention

All 4 studies [24-27] investigated educational interventions that used clinical simulation mediated by XR technologies, with the aim of developing clinical competencies in obstetric or immediate neonatal care.

The simulated clinical contexts included: preparation of emergency tocolytics and preparation of the pregnant woman for cesarean section [24]; eutocic delivery, dystocia, and umbilical cord prolapse [26]; immediate newborn care [25]; and neonatal asphyxia and resuscitation [24,26,27]. Neonatal care was a common component in all 4 programs analyzed, consistently simulated in the context of immediate newborn care, immediately after birth.

Of the 4 studies included, 3 presented multiple simulation scenarios within the same intervention using VR [26,27] or AR [24]. None of the included studies provided detailed information about the physical simulated environment used in the interventions.

### Technology Used

The included interventions used technically distinct XR configurations, with VR being more common than AR. Based on the operational definition used in this review, 3 studies were classified as VR because they used headset-based immersive delivery and first-person task-based interaction within simulated clinical scenarios [25-27]. Two of these used head-mounted display (HMD)–based VR with handheld controllers [25,26], while one used a low-cost smartphone-based HMD with a VR application [27]. One study was classified as AR because it used a smartphone-based marker system to overlay and manipulate virtual elements within the real-world environment [24]. The main technical characteristics of the interventions are summarized in Table 2.

Across studies, interaction was generally described as first-person and task-based, requiring participants to perform clinical actions or manipulate virtual elements within simulated obstetric or neonatal scenarios [24-27]. However, the level of interactivity was not consistently detailed. In particular, information on free navigation, branched decision-making, customization of the virtual environment, and learner control was absent or limited.

Reporting of exposure and technical design features was also incomplete. Although some studies reported session duration or total usage time, the number of sessions and degree of learner autonomy were often unclear. Similarly, visual fidelity, spatial realism, software licensing, reusability, and open-access availability were not described. This limited reporting restricts comparison of the technological complexity and pedagogical affordances of the XR systems used across studies.

**Table 2.** Technical characteristics of XR^a^ interventions.

Study	XR type	Device (hardware)	Software	Interaction	Duration and exposure	Presence and usability	Adverse effects
Vogel et al (2024) [24]	AR^b^	Smartphone +marker	Heb@AR	Active task-based interaction through manipulation of the virtual elements	Not reported	Not reported	Not reported
Öner and Turfan (2024) [25]	VR^c^	HMD^d^ (Oculus Quest)+handheld controllers	Unity-based VR simulator	Active task-based interaction through manipulation of the virtual environment	Up to 10 minutes per session	Presence assessed; Usability not reported.	Dizziness and nausea
Zhao et al (2024) [26]	VR	HMD+handheld controllers	CBL-VR^e^ platform	Active task-based interaction through manipulation of the virtual environment and feedback	Not reported	Not reported	Mild cybersickness (not quantified)
Umoren et al (2021) [27]	VR	Low-cost HMD+smartphone	eHBB^f^ VR app	Active task-based interaction through manipulation of the virtual environment	101 minutes (IQR 81‐120) over 6-month follow-up	Usability and acceptability assessed; presence not reported.	Not reported

a XR: extended reality.

b AR: augmented reality.

c VR: virtual reality.

d HMD: head-mounted display.

e CBL-VR: case-based learning virtual reality.

f eHBB: electronic Helping Babies Breathe.

### Theoretical and Pedagogical Frameworks

Two studies [25,26] reported an explicit pedagogical or theoretical basis for their immersive simulation interventions. Zhao et al [26] integrated case-based learning (CBL) with VR simulation to support clinical reasoning, while Öner and Turfan [25] referred to the Jeffries Simulation Theory [28] as a framework for the design, implementation, and evaluation of the simulation. The remaining studies did not report an explicit theoretical model or instructional framework [24,27].

Across the included studies, the interventions primarily targeted the development of technical competencies related to obstetric or neonatal care, particularly procedures associated with childbirth, obstetric emergencies, immediate newborn care, and neonatal resuscitation [24-27]. Nontechnical or behavioral competencies were less frequently addressed; only Zhao et al [26] explicitly included communication, teamwork, and clinical decision-making.

Although Bloom's Taxonomy [18] was not explicitly used by the primary studies, the described activities could be mapped mainly to the cognitive and psychomotor domains. All studies involved the application of knowledge in simulated clinical contexts and the performance of obstetric or neonatal skills [24-27]. Higher-order cognitive processes, such as case analysis and decision-making, as well as affective elements related to teamwork, were identified only in Zhao et al [26].

Similarly, none of the studies explicitly adopted the Kirkpatrick model [19] as an evaluative framework. However, based on the reported outcomes, all studies assessed level 2 outcomes, including knowledge, practical skills, and/or attitudes. Three studies also assessed level 1 outcomes, such as satisfaction or perceptions of the learning experience [24-26]. No study evaluated level 3 outcomes or level 4 outcomes.

Reporting of instructional design and simulation best-practice components was limited. None of the studies described the use of a structured instructional design model, although 2 cited the Healthcare Simulation Standards of Best Practice [29] as a guiding reference for simulation development and implementation [24,25]. Structured pedagogical briefing was not clearly reported. Instead, most studies described technical orientation to the technology, such as guidance on the use of VR or AR devices [24,26,27].

Debriefing was also inconsistently described. Only Öner and Turfan [25] reported a structured debriefing process, supported by the Jeffries Simulation Theory [28] and based on audiovisual review of the simulation. Other studies described less structured forms of feedback, including automated feedback provided by the application [24], feedback during the activity [26,27], or facilitator-mediated group feedback after the activity [26].

In all included studies, simulation was conducted individually. In one study, the facilitator was described as playing an active role providing guided instruction during the simulation [25]; in another, the activity was conducted without supervision, characterizing autonomous simulation [27]. In the remaining studies, no information was provided about the facilitator’s role during the simulation [24,26].

Formal validation of scenario design was not reported in any study. One study used a validated clinical algorithm to structure the expected sequence of actions [27], while others reported that scenarios were based on clinical practice situations and reviewed by faculty members or experts [25,26].

Curricular integration was explicitly described only by Vogel et al [24], who used AR simulation as a complement to theoretical and practical obstetric teaching. Other studies did not specify whether the interventions were embedded in formal curricula. Across studies, immersive simulation was combined with additional teaching strategies, including preparatory reading, digital guide, lectures, mannequin-based teaching, clinical skills training, team-based role-play, and digital learning materials [24-27].

### Instruments and Assessment

The included studies used different assessment instruments, which were distributed across the domains of knowledge, skills, and attitudes, in addition to addressing aspects related to the learning experience.

Theoretical knowledge was assessed using instruments constructed by the authors themselves based on the objectives of the intervention [24] or developed within the context of international educational programs [27].

Clinical skills were evaluated through checklists applied during the simulation [25,26], in Objective Structured Clinical Examination (OSCE) stations [27], and through online questionnaires of self-perceived practical performance [24].

Attitude was assessed in 2 studies, through validated self-administered scales that measured self-confidence [25] and self-directed learning ability [26].

Learning experiences and perceptions of the simulated environment were addressed differently across studies. Only Öner and Turfan [25] used validated scales to measure satisfaction, immersion perception, scenario design, and pedagogical practices. In the others, nonvalidated questionnaires were applied to assess aspects such as satisfaction [26], usability, realism, and clinical applicability [27].

### Outcomes of the Educational Interventions

The included studies reported outcomes related to knowledge, practical skills, attitudes, and learning experience. Overall, findings suggested positive effects of VR- and AR-based interventions, particularly for practical skills, self-confidence, satisfaction, and perceived learning. However, the heterogeneity of outcome measures, assessment instruments, and follow-up periods limited direct comparison across studies.

Knowledge outcomes were assessed in 2 studies [24,27], with mixed findings. Vogel et al [24] reported significant improvements in perceived knowledge after AR simulation across all simulated clinical scenarios. In contrast, Umoren et al [27] found no statistically significant differences between groups in theoretical knowledge following an in-person course, although participants had only been exposed to the VR application for technical familiarization at that stage.

Practical skills were assessed in all 4 studies. Three studies reported significant improvements or higher performance associated with immersive simulation [24-26]. The strongest differences were observed when VR was combined with CBL, particularly for team-based skills [26], and when VR was compared with low-fidelity simulation for newborn care training [25]. In contrast, Umoren et al [27] did not identify statistically significant differences between groups in immediate or long-term skills performance, although the VR group showed a trend toward better retention at follow-up. Detailed findings for practical skills are presented in Table 3. As Vogel et al [24] did not include a control group, their results were not included in this analysis.

Attitudinal outcomes were assessed in 3 studies. Improvements were reported in self-confidence after AR simulation [24] and VR simulation [25], while Zhao et al [26] found gains in self-directed learning, including self-management, information processing, and collaboration, compared with traditional lecture-based teaching.

Learning experience was generally evaluated positively across studies. Participants reported favorable perceptions of satisfaction, usability, realism, clinical usefulness, immersion, and educational value [24-27]. However, validated instruments were used inconsistently, limiting comparison across interventions. Reported implementation challenges included mobile device compatibility issues, AR marker detection failures, dependence on project-provided smartphones, low adherence to recommended practice, and symptoms such as dizziness, nausea, or mild cybersickness [24-27].

**Table 3.** Instruments for data collection and observed outcomes related to the practical skills domain in the studies included in the systematic review.

Study and measurement instrument	Outcome of interest	Control group	Intervention group	Mean difference^a^	*P* value	Effect size (Cohen *d*)
Zhao et al [26]						
Academic performance evaluation form	Individual skill: normal birth, dystocia, umbilical cord prolapse, and neonatal resuscitation	89.24 (3.15)^b^	90.88 (2.14)^b^	+1.64	<.001	0.63
Academic performance evaluation form	Team-based skill: normal birth, dystocia, umbilical cord prolapse, and neonatal resuscitation	81.28 (5.45)^b^	90.97 (2.33)^b^	+9.69	<.001	2.42
Academic performance evaluation form	Case analysis: normal birth, dystocia, umbilical cord prolapse, and neonatal resuscitation	86.70 (2.56)^b^	88.64 (3.19)^b^	+1.94	<.001	0.66
Öner and Turfan [25]						
Practice skills list	Initial newborn care	74.17 (13.55)^b^	83.28 (19.67)^b^	+9.11	.01	0.54
Umoren et al [27]						
OSCE^c^ A	Routine newborn care and initial resuscitation	72%^d^	76%^d^	+3.8%	.61	—^e^
OSCE B	Prolonged neonatal resuscitation	49%^d^	62%^d^	+13.2%	.09	—^e^
BMV^f^ Skill check	Bag-mask ventilation in the newborn	22%^d^	28%^d^	+5.5%	.48	—^e^

a Mean difference represents the difference between the intervention and control groups.

b Mean (SD) of students’ scores (scores ranging from 0‐100).

c OSCE: Objective Structured Clinical Examination.

d Pass rate (%). Percentages are reported as presented in the original studies.

e Effect size not reported in the original study.

f BMV: bag-and-mask ventilation.

### Methodological Quality and Risk of Bias

Overall, the included studies presented low to moderate risk of bias, with methodological limitations mainly related to blinding, allocation concealment, outcome measurement, and control of confounding factors. These limitations are common in educational intervention research but should be considered when interpreting the findings.

The 2 randomized controlled trials reported randomization, baseline comparability between groups, appropriate outcome assessment methods, and adequate statistical analyses [25,27]. However, allocation concealment was not described, and blinding of participants, intervention providers, or outcome assessors was not implemented. In addition, Öner and Turfan [25] did not report an intention-to-treat analysis, and the use of self-assessment measures may have increased the risk of measurement bias.

The 2 quasi-experimental studies reported a clear temporal relationship between intervention and outcomes, complete follow-up, and appropriate statistical analysis [24,26]. However, only Zhao et al [26] included a control group and reported baseline similarity between groups. In both quasi-experimental studies, limited information was provided on strategies to standardize data collection or ensure consistency among assessors. Vogel et al [24], which used a single-group pre-post design, did not report procedures to control for potential cointerventions.

MERSQI scores ranged from 10.0 to 14.5, with a mean score of 12.9 (SD 2.0), indicating moderate methodological quality overall. Higher scores were associated with stronger study designs, objective outcome measures, and appropriate statistical analyses. Lower scores mainly reflected limited evidence of instrument validity, single-institution sampling in most studies, and the absence of higher-level educational outcomes. Detailed MERSQI scores are presented in Multimedia Appendix 2.

Given the small number of included studies and the heterogeneity in interventions, outcome domains, assessment instruments, and timing of evaluation, a formal GRADE (Grading of Recommendations Assessment, Development, and Evaluation) assessment was not conducted. Instead, the overall confidence in the evidence base was assessed narratively, considering study design, risk of bias (JBI), methodological quality (MERSQI), consistency and directness of findings, and the use of validated outcome measures. Reporting bias could not be formally assessed using statistical methods, such as funnel plots, due to the limited number of studies and the absence of pooled effect estimates.

## Discussion

### Overview

This systematic review examined how VR and AR have been integrated into obstetric nursing and midwifery education, with particular attention to theoretical grounding, instructional design, technological characteristics, and educational outcomes. The findings indicate that immersive technologies have been used mainly to support the development of technical competencies in obstetric and neonatal care, particularly in childbirth procedures, emergency scenarios, immediate newborn care, and neonatal resuscitation. However, the evidence base remains limited, consisting of only 4 heterogeneous studies, and the pedagogical and technological reporting of the interventions was often incomplete.

In general, the included studies suggest that VR- and AR-based interventions may improve immediate learning outcomes, particularly practical skills, self-confidence, satisfaction, and perceived learning. However, the incorporation of theoretical frameworks and instructional design was limited, and these findings should therefore be interpreted with caution.

### Theoretical and Pedagogical Grounding

A central finding of this review was the limited explicit use of theoretical or pedagogical frameworks.

Only one study referred to the Jeffries Simulation Theory [28], and another integrated CBL as a pedagogical strategy [26]. The remaining studies did not clearly describe the theoretical assumptions guiding the design, implementation, or evaluation of the XR-based interventions. This is relevant because, in technology-enhanced simulation-based learning, conceptual clarity is essential to strengthen pedagogical coherence, methodological rigor, and reproducibility [5]. Theories provide the conceptual foundation for selecting instructional strategies and organizing learning experiences according to learners’ prior knowledge, motivation, and educational needs [30,31], while the instructional models translate these theoretical foundations into practical teaching strategies.

This finding is consistent with previous reviews showing that the absence or superficial use of theoretical frameworks remains a recurrent limitation in simulation and immersive technology research in nursing education [9,10,32]. Although constructivist and experiential approaches, such as Kolb Experiential Learning Theory [33], are frequently cited in VR simulation and technology-mediated nursing education [10,34], the studies included in this review rarely made explicit how learning theory informed scenario design, facilitation, feedback, or assessment. As a result, the interventions often appeared primarily technology, or procedure, driven rather than theory-informed.

Recent models specific to immersive learning, including the Cognitive Affective Model of Immersive Learning (CAMIL) [35], the Immersive Virtual Reality Pedagogical Model (iVRPM) [36], and embodied approaches to immersive extended reality [37], are useful for interpreting this gap. These models highlight constructs such as presence, agency, interactivity, feedback, embodiment, and cognitive engagement as mechanisms through which XR may support learning. However, in the included studies, these mechanisms were seldom assessed or clearly operationalized. For example, presence was measured in only one study [25], while constructs such as agency, cognitive load, usability, embodiment, and learner control were either not evaluated or insufficiently reported. Therefore, although these models offer relevant conceptual guidance, their application in obstetric nursing and midwifery XR education remains limited and requires further empirical validation.

The review also identified a lack of nursing or midwifery theoretical models oriented toward clinical practice. None of the included studies incorporated frameworks addressing comprehensive, relational, woman- and family-centered care, despite the relevance of these dimensions to nurse-midwives’ professional practice [38]. This omission may contribute to a predominantly technicist approach to XR-based education, focused mainly on procedural performance and emergency management, while giving less attention to communication, emotional support, shared decision-making, and professional values [39]. Continuous exposure to the theoretical foundations of the profession can help students recognize how these principles apply in real care contexts [40] and may support patient-centered decision-making and therapeutic interventions [41,42]. Nevertheless, future XR interventions in obstetric nursing and midwifery should more explicitly integrate disciplinary theories to ensure that immersive simulation supports not only technical competence but also holistic and person-centered care.

### Instructional Design Models

Instructional design was another area with limited explicit reporting across the included studies. Although some interventions incorporated pedagogical strategies such as CBL, repeated practice, preparatory activities, and feedback, none clearly described the instructional principles or models guiding the organization, sequencing, or progression of the learning experience. This limits the interpretation of how VR or AR was integrated into the educational process and reduces the replicability of the interventions in other contexts [30].

This gap is relevant because immersive technologies should not function as isolated or decontextualized learning activities, but as components of a broader instructional plan aligned with learning objectives, learner characteristics, and expected outcomes [30,43] as their effectiveness depends on the quality of instructional design [44]. Instructional design models such as Gagné's 9 events of instruction [45], Analysis, Design, Development, Implementation, and Evaluation (ADDIE)[46], and Four-Component Instructional Design (4C/ID) [47] have been successfully applied in health and nursing education, including in obstetric emergency training and complex educational programs [48-50]. However, none of the studies included in this review explicitly adopted these or other structured instructional design models.

Although 2 studies [24,25] reported using the International Nursing Association for Clinical Simulation and Learning (INACSL) Healthcare Simulation Standards of Best Practice [29], their operationalization was only partially described, with key components such as learning objectives, briefing, facilitation, scenario design, feedback, and debriefing inconsistently reported.

The mapping of the interventions to Bloom's Taxonomy [18] showed that most studies addressed cognitive and psychomotor domains, mainly through the application of knowledge and performance of clinical procedures. However, the taxonomy was not explicitly used in instructional planning. This limits understanding of whether the degree of immersiveness and interactivity was intentionally aligned with different levels of cognitive complexity, as recommended in technology-mediated learning environments [36,51]. Similarly, although the Kirkpatrick model [19] was useful for interpreting evaluation levels, it was not explicitly adopted by the primary studies. All studies assessed learning outcomes, and some assessed satisfaction or perceptions, but none evaluated transfer to clinical practice or organizational or patient-related outcomes, reflecting a recurrent limitation in health professions education research [52].

The limited description of preparatory activities, briefing, and debriefing is particularly important in XR-based simulation. From the perspective of Cognitive Load Theory [53], these components may help manage the cognitive demands of immersive environments by preparing learners for the task, reducing unnecessary ambiguity, and supporting reflection after the experience. All studies described some form of preparation, such as theoretical classes, preparatory reading, technical instructions, practical training, or mannequin-based activities [24,26]. These strategies may have helped students build prior knowledge and reduce task complexity [31,54]. However, structured pedagogical briefing was rarely reported; most studies focused on technical familiarization with VR or AR devices rather than on broader briefing elements such as learning objectives, roles, expectations, psychological safety, confidentiality, and the fiction contract [10,55].

Debriefing was also insufficiently described. Only one study reported a structured debriefing process supported by a theoretical framework [25]. Another combined automated feedback with facilitator-guided discussion, suggesting a potentially useful hybrid approach [26]. Evidence indicates that debriefing and immediate feedback can strengthen clinical competence in VR simulation by supporting reflection, repetition, and individualized learning [56]. Automated feedback may therefore have pedagogical value when it is intentionally designed and aligned with learning objectives, but it should not be considered a substitute for facilitator-led reflective debriefing [57].

### Pedagogical Implications of Immersiveness and Interactivity in Learning

The reviewed interventions used immersive and interactive technologies to support experiential learning in obstetric and neonatal scenarios. VR interventions generally relied on HMDs to create 3D environments, while the AR intervention used smartphone-based interaction. These features may enhance presence, realism, active participation, and knowledge retention when they are pedagogically aligned with the learning objectives [35,36,58].

However, the included studies provided limited information on how the level and type of immersion were selected or justified. In simulation-based education, immersion should not be understood only as physical or technological realism. Rather, different dimensions of fidelity may support different learning goals. Physical fidelity refers to the extent to which the simulation resembles the real clinical environment in sensory or material terms, whereas functional fidelity concerns the extent to which the simulation reproduces the relevant clinical tasks, decision pathways, and responses. Psychological fidelity refers to the extent to which the scenario engages learners cognitively and emotionally, encouraging them to think and act as they would in clinical practice. In this sense, high physical realism may be useful for spatial orientation, procedural familiarization, and anatomical understanding, while functional and psychological fidelity may be more important for clinical reasoning, diagnostic decision-making, communication, and prioritization in complex obstetric situations [59-61].

This distinction is particularly relevant for XR-based obstetric education. For lower-level cognitive objectives, such as remembering or understanding, highly immersive or highly interactive environments may not be necessary. Greater interactivity and psychological engagement may be more appropriate when learners are expected to apply knowledge, analyze clinical cues, make decisions, or coordinate care in emergency scenarios [38]. Thus, the educational value of VR and AR depends less on maximizing realism in all dimensions and more on aligning the type of fidelity with the intended competencies.

The findings also suggest that immersive design should be considered in relation to cognitive load. High levels of sensory stimulation, realism, or interaction may support engagement, but they may also increase extraneous load if learners are insufficiently prepared or if interface complexity distracts from the clinical task [11]. Although some studies described technical familiarization or preparatory activities, none explicitly reported how physical, functional, or psychological fidelity, interface design, or task complexity were adjusted to learner proficiency or cognitive demands.

Among the studies analyzed, only Öner and Turfan [25] assessed the sense of presence, reporting high perceived immersion and a positive relationship with students’ performance and satisfaction. Zhao et al [26] also reported positive perceptions related to satisfaction and self-confidence. However, Umoren et al [27] found low adherence over time despite good usability, suggesting that ease of use and physical immersion alone may not ensure sustained engagement or motivation [58].

Although all studies described first-person interaction and the performance of clinical actions in 3D environments, none detailed the technical characteristics of the systems used, such as visual fidelity, navigation freedom, or decision-making mechanisms. Conversely, research indicates that these features of VR and AR are particularly effective for teaching complex anatomical and spatial content, with positive results also observed in obstetrics, especially due to the ability to visualize the dynamic relationship between the fetus and the maternal pelvis [1,3,8]. In addition, the incorporation of haptic resources and tactile, auditory, or visual feedback can enhance the sense of realism and encourage learners to reflect on their actions, thereby fostering the development of clinical reasoning. However, when poorly calibrated, these additional stimuli may cause distraction or sensory overload [11].

The technical diversity of the included interventions also indicates that the findings should not be interpreted as reflecting a homogeneous “XR effect.” HMD-based VR, smartphone-based HMD VR, and smartphone-based AR differ in the type and degree of immersion they provide, as well as in their potential to support presence, spatial understanding, embodied interaction, and learner control. Therefore, as immersive technologies continue to expand in health professions education, there is a growing need to better understand the mechanisms through which learning occurs in these environments. Beyond the assessment of learning outcomes, future research should incorporate key constructs such as presence, agency, and cognitive load, assessed using validated instruments (eg, the Multidimensional Cognitive Load Scale for Virtual Environments [62], the Presence Questionnaire [63], and NASA-TLX [64])**,** as well as usability measures (eg, virtual reality system usability questionnaire [65]), to provide a more comprehensive understanding of XR-based learning processes.

### Educational Effects of VR- and AR-Based Interventions

The included studies reported generally favorable educational outcomes for VR- and AR-based interventions, particularly in relation to practical skills, self-confidence, satisfaction, and perceived learning. However, these findings should be interpreted cautiously because the evidence base was small, heterogeneous, and mainly limited to short-term educational outcomes.

Assessment strategies varied considerably across studies, with a predominance of measures related to technical skills, learning experience, and perceived self-confidence. Most studies assessed outcomes immediately after the intervention, limiting conclusions about retention and transfer to clinical practice. Only Umoren et al [27] included follow-up assessment, suggesting a possible benefit of VR for the retention of psychomotor competencies. However, the absence of longitudinal assessment in the remaining studies prevents broader conclusions about the sustainability of learning effects.

The reviewed studies were concentrated at Kirkpatrick level 2, as they assessed learning outcomes such as knowledge, skills, and attitudes. Some studies also addressed level 1 outcomes, including satisfaction and perceptions of the learning experience. However, none evaluated level 3 (behavioral change in clinical practice) or level 4 outcomes (organizational, clinical, or patient-level impact) [19]. This distinction is important because improvements observed in simulated learning environments should not be interpreted as direct evidence of improved clinical performance or patient outcomes. Capturing higher-level Kirkpatrick outcomes is particularly challenging in obstetric emergency education. Events such as neonatal resuscitation, dystocia, umbilical cord prolapse, or emergency cesarean preparation may be high-risk but relatively infrequent, making it difficult to observe sufficient real-world cases after training. This limitation is consistent with previous reviews on virtual reality [66] and reflects methodological challenges inherent to clinical training, where assessing behavioral change in real practice and linking training to patient outcomes require longitudinal follow-up and are influenced by multiple confounding factors [52]. This may result in imprecise evidence, as observed in reviews of simulation in obstetric emergencies [67], reinforcing the need for caution when interpreting and measuring these outcomes.

Most interventions focused primarily on psychomotor skills and basic cognitive outcomes, especially the understanding and application of technical procedures. This focus is consistent with previous evidence suggesting that immersive technologies may support cognitive, psychomotor, and affective outcomes among nursing students and nurses [2,68], with promising applications in obstetric education [69]. However, higher-order cognitive processes were less consistently addressed. Zhao et al [26] was the only study that clearly stimulated clinical reasoning and decision-making through a CBL approach, integrating case analysis, diagnosis formulation, and procedural execution. This finding aligns with evidence associating VR with critical thinking and cognitive engagement among nursing students when it is embedded in intentional instructional design [12,70].

Despite the promising results in the cognitive and psychomotor domains, Zhao et al [26] was the sole study to address aspects of communication and teamwork, which were evaluated in the context of collaborative clinical case resolution. Recent evidence broadens this discussion by demonstrating that the use of immersive virtual environments can promote significant gains in communication, leadership, situational awareness, teamwork, and decision-making [71], particularly when there is a high degree of realism and interactivity [5,71]. These are fundamental competencies for safe clinical performance in obstetric contexts. Nevertheless, the literature indicates that the development and structured assessment of these competencies in VR and AR–based programs remain incipient, representing an important challenge for future research in obstetric and nursing education [32].

The included studies predominantly focus on procedural skills, with limited attention to the relational dimensions of care, revealing an important imbalance in midwifery training, which requires the integration of clinical, emotional, and interpersonal components [38].

### Implications and Recommendations for Educational Practice

The integration of simulation mediated by VR and AR in obstetric education requires coherence among theoretical foundations, instructional design, technological features, and expected outcomes. Immersive technologies should be used as pedagogical tools within a structured educational program, rather than as isolated technological experiences. This recommendation is consistent with the recent Utstein-style consensus agenda for XR in health care simulation, which emphasizes evidence-informed adoption, faculty readiness, institutional investment, data-driven evaluation, and sustainable integration of XR technologies in health professions education [72].

Considering the context of obstetric nursing and midwifery education, nursing theories can guide the principles of comprehensive and person-centered care, reinforcing the ethical and disciplinary commitments of the nursing and midwifery professions. These values should permeate the construction and implementation of simulation scenarios, ensuring that students develop clinical judgment, ethical awareness, and decision-making grounded in evidence and in the needs expressed by women and their families.

In instructional planning, it is recommended that VR and AR be integrated into the curriculum as part of a continuum connecting theory, simulated practice, and clinical practice. To achieve this, it is suggested to use instructional design models that intentionally and systematically integrate different teaching strategies, including immersive technologies, grounded in the articulation between pedagogical theory and the expected learning outcomes.

The level of immersiveness and interactivity should be adjusted according to the cognitive levels involved and the learners’ proficiency, balancing realism and cognitive load. Less immersive environments are indicated when the focus is on understanding concepts and initial procedural practice, whereas highly immersive simulations are recommended for learning objectives involving clinical reasoning and decision-making in complex obstetric contexts. Furthermore, obstetric education can leverage the potential of VR and AR to represent dynamic anatomical and physiological structures, such as the relationship between the fetus and the maternal pelvis during labor, thereby enhancing spatial understanding and clinical reasoning, provided that the simulation design avoids sensory distractions and maintains intentionality in all elements of the scenario.

Clinical simulation mediated by immersive technologies should follow international guidelines for best practices in clinical simulation, ensuring both pedagogical and ethical quality. Preliminary preparation is recommended to reduce intrinsic cognitive load through familiarization with the technology and the learning content. It is important to consider briefing as an essential stage for reducing extraneous cognitive load by aligning objectives and expectations, while facilitation should monitor and adjust cognitive load during the simulation, providing support compatible with the learner’s proficiency level and encouraging autonomy.

Automated sensory feedback from virtual platforms can be useful for validating actions or signaling the need for adjustments in clinical reasoning, and when complemented by facilitator-mediated feedback, it promotes reflective learning and deeper understanding. During debriefing, conducted in a structured manner, it is important to include reflection on technical and behavioral dimensions related to woman- and family-centered care.

The evaluation of simulations mediated by VR or AR should encompass multiple dimensions and outcome levels, in alignment with the New World Kirkpatrick Model [19] and the cognitive, psychomotor, and affective domains. Evaluation should occur at different points in time, monitoring the retention and transfer of skills to obstetric care settings, and the results should provide feedback for the continuous improvement of the design, facilitation, and curricular integration of immersive technologies.

### Limitations of the Review Process

In addition to the limitations of the included studies, some limitations of the review process should be acknowledged. The small number of included studies and their substantial heterogeneity limited direct comparisons across interventions. Although the included studies reported generally favorable educational outcomes, the evidence reflects considerable variation in technologies, pedagogical approaches, outcome measures, and assessment time points.

Furthermore, the synthesis was constrained by the information reported in the primary studies, precluding formal assessment of reporting bias and certainty of evidence. Consequently, the overall confidence in the evidence is limited. Most outcomes were restricted to satisfaction, perceived learning, knowledge, or skills, with no evidence on transfer to clinical practice or impact on organizational or patient outcomes.

Accordingly, these findings should be interpreted as preliminary, despite their potential implications for midwifery and obstetrics education.

### Conclusion

Evidence suggests that VR and AR may support learning in obstetric nursing and midwifery education, particularly in relation to practical skills, self-confidence, satisfaction, and perceived learning. However, the overall certainty of the evidence remains limited because of the small number of heterogeneous studies.

Stronger integration of learning theories, instructional design models, simulation best-practice standards, and validated assessment strategies is needed. Future XR-based interventions should be pedagogically intentional, transparently reported, and aligned not only with technical competence but also with the relational, ethical, and woman-centered nature of obstetric and midwifery care.

## Supplementary material

10.2196/90252 Multimedia Appendix 1 Detailed search strategy and eligibility criteria used in the systematic review.

10.2196/90252 Multimedia Appendix 2 Risk-of-bias assessment of included studies.

10.2196/90252 Checklist 1 PRISMA 2020 checklist for the systematic review.

## Abbreviations

4C/ID: Four-Component Instructional Design
ADDIE: Analysis, Design, Development, Implementation, and Evaluation
AR: augmented reality
CAMIL: Cognitive affective model of immersive learning
CBL: case-based learning
CINAHL: Cumulative Index to Nursing and Allied Health Literature
GRADE: Grading of Recommendations Assessment, Development, and Evaluation
HMD: head-mounted display
INACSL: International Nursing Association for Clinical Simulation and Learning
iVRPM: Immersive Virtual Reality Pedagogical Model
JBI: Joanna Briggs Institute
MERSQI: Medical Education Research Study Quality Instrument
OSCE: Objective Structured Clinical Examination
PIO: population, intervention, outcome
PRISMA: Preferred Reporting Items for Systematic Reviews and Meta-Analyses
PROSPERO: International Prospective Register of Systematic Reviews
RCT: randomized controlled trial
SWiM: Synthesis Without Meta-Analysis
VR: virtual reality
XR: extended reality

## References

[1] Aasekjær K, Bjørnås B, Skivenes HK, Vik ES (2024). Immersive virtual reality (VR) when learning anatomy in midwifery education: a pre-post pilot study. Eur J Midwifery.

[2] Efendi D, Apriliyasari RW, Prihartami Massie JGE (2023). The effect of virtual reality on cognitive, affective, and psychomotor outcomes in nursing staffs: systematic review and meta-analysis. BMC Nurs.

[3] Jallad ST, Natsheh I, Helo LA (2024). Nursing student’s perceptions, satisfaction, and knowledge toward utilizing immersive virtual reality application in human anatomy course: quasi-experimental. BMC Nurs.

[4] Hamilton D, McKechnie J, Edgerton E, Wilson C (2021). Immersive virtual reality as a pedagogical tool in education: a systematic literature review of quantitative learning outcomes and experimental design. J Comput Educ.

[5] Stenseth HV, Steindal SA, Solberg MT (2025). Simulation-based learning supported by technology to enhance critical thinking in nursing students: scoping review. J Med Internet Res.

[6] Lin XP, Li BB, Yao ZN, Yang Z, Zhang M (2024). The impact of virtual reality on student engagement in the classroom-a critical review of the literature. Front Psychol.

[7] Hasyim AR, Syarif S, Ahmad M, Niswar M, Nasrudin AM (2021). Enhance midwifery student skills about active management third stage labor via learning media. Gac Sanit.

[8] Ryan G, Callaghan S, Rafferty A (2022). Virtual reality in midwifery education: a mixed methods study to assess learning and understanding. Nurse Educ Today.

[9] Hernon O, McSharry E, MacLaren I, Dunne R, Carr PJ (2023). The use of educational technology in undergraduate and postgraduate nursing and midwifery education: a scoping review. Comput Inform Nurs.

[10] Koskinen I, Stolt M, Widmer CT (2024). Methodological approaches and competence areas of nursing students in virtual reality simulation research - a scoping review. Nurse Educ Today.

[11] Parong J, Mayer RE (2021). Cognitive and affective processes for learning science in immersive virtual reality. Comput Assist Learn.

[12] Sim JJM, Rusli KDB, Seah B, Levett-Jones T, Lau Y, Liaw SY (2022). Virtual simulation to enhance clinical reasoning in nursing: a systematic review and meta-analysis. Clin Simul Nurs.

[13] Asoodar M, Janesarvatan F, Yu H, de Jong N (2024). Theoretical foundations and implications of augmented reality, virtual reality, and mixed reality for immersive learning in health professions education. Adv Simul (Lond).

[14] Bauce K, Kaylor MB, Staysniak G, Etcher L (2023). Use of theory to guide integration of virtual reality technology in nursing education: a scoping study. J Prof Nurs.

[15] (2024). JBI Manual for evidence synthesis. JBI.

[16] Page MJ, McKenzie JE, Bossuyt PM (2021). The PRISMA 2020 statement: an updated guideline for reporting systematic reviews. BMJ.

[17] Ouzzani M, Hammady H, Fedorowicz Z, Elmagarmid A (2016). Rayyan-a web and mobile app for systematic reviews. Syst Rev.

[18] Anderson LW, Krathwohl DR (2001). A taxonomy for learning, teaching, and assessing: a revision of bloom’s taxonomy of educational objectives.

[19] Kirkpatrick JD, Kirkpatrick WK (2016). Kirkpatrick’s Four Levels of Training Evaluation: Association for Talent Development.

[20] Barker TH, Stone JC, Sears K (2023). The revised JBI critical appraisal tool for the assessment of risk of bias for randomized controlled trials. JBI Evid Synth.

[21] Barker TH, Habibi N, Aromataris E (2024). The revised JBI critical appraisal tool for the assessment of risk of bias for quasi-experimental studies. JBI Evid Synth.

[22] Cook DA, Reed DA (2015). Appraising the quality of medical education research methods: the medical education research study quality instrument and the Newcastle-Ottawa Scale-education. Acad Med.

[23] Campbell M, McKenzie JE, Sowden A (2020). Synthesis without meta-analysis (SWiM) in systematic reviews: reporting guideline. BMJ.

[24] Vogel K, Bernloehr A, Willmeroth T, Blattgerste J, Hellmers C, Bauer NH (2024). Augmented reality simulation-based training for midwifery students and its impact on perceived knowledge, confidence and skills for managing critical incidents. Midwifery.

[25] Öner S, Turfan EÇ (2024). Virtual reality versus low level simulation in newborn care teaching in Turkey. Afr J Reprod Health.

[26] Zhao L, Dai X, Chen S (2024). Effect of the case-based learning method combined with virtual reality simulation technology on midwifery laboratory courses: a quasi-experimental study. Int J Nurs Sci.

[27] Umoren R, Bucher S, Hippe DS (2021). eHBB: a randomised controlled trial of virtual reality or video for neonatal resuscitation refresher training in healthcare workers in resource-scarce settings. BMJ Open.

[28] Jeffries PR (2016). The NLN Jeffries Simulation Theory.

[29] Watts PI, McDermott DS, Alinier G (2021). Healthcare simulation standards of best practice simulation design. Clin Simul Nurs.

[30] Frèrejean J, Walker K, Symon B, Eppich W (2025). Insights from 40 years of educational research: honoring Jeroen van Merriënboer. Adv Simul (Lond).

[31] O’Leary F (2024). How to deliver effective paediatric simulation based education. Paediatr Respir Rev.

[32] Ropponen P, Tomietto M, Pramila-Savukoski S (2025). Impacts of VR simulation on nursing students’ competence, confidence, and satisfaction: a systematic review and meta-analysis of randomised controlled trials. Nurse Educ Today.

[33] Kolb DA (1984). Experiential Learning: Experience as the Source of Learning and Development.

[34] O’Connor S, Kennedy S, Wang Y, Ali A, Cooke S, Booth RG (2022). Theories informing technology enhanced learning in nursing and midwifery education: a systematic review and typological classification. Nurse Educ Today.

[35] Makransky G, Petersen GB (2021). The Cognitive Affective Model of Immersive Learning (CAMIL): a theoretical research-based model of learning in immersive virtual reality. Educ Psychol Rev.

[36] Bicalho DR, Piedade J, Matos JF (2025). iVRPM: Conceptual proposal of an immersive virtual reality pedagogical model. Appl Sci.

[37] Fugate JMB, Tonsager MJ, Macrine SL (2025). Immersive extended reality (I-XR) in medical and nursing for skill competency and knowledge acquisition: a systematic review and implications for pedagogical practices. Behav Sci (Basel).

[38] (2024). Transitioning the midwifery models of care: global position paper.

[39] Cruz M da S, Denucci MAM (2025). Teaching principles and values in nursing from a philosophical perspective of care [Article in Portugese]. Rev Foco.

[40] Norris Waller M, Newsome Wicks M (2021). Is there still value in teaching nursing theory?. J Nurs Educ.

[41] Bilgiç FŞ, Bozkurt G (2024). Effect of online infant care training and postpartum counseling based on Meleis’ transition theory on mothers’ readiness for care and breastfeeding: a randomized controlled trial. Clin Exp Pediatr.

[42] Estakhri RK, Ezbarami ZT, Peyrovi H (2025). Effectiveness of transition theory-based ointerventions on outcomes of nursing care: a scoping review. Iran J Nurs Midwifery Res.

[43] Mallek F, Mazhar T, Faisal Abbas Shah S, Ghadi YY, Hamam H (2024). A review on cultivating effective learning: synthesizing educational theories and virtual reality for enhanced educational experiences. PeerJ Comput Sci.

[44] Cook DA, Hamstra SJ, Brydges R (2013). Comparative effectiveness of instructional design features in simulation-based education: systematic review and meta-analysis. Med Teach.

[45] Gagné RM (1985). The Conditions of Learning and Theory of Instruction.

[46] Branch RM (2009). Instructional Design: The ADDIE Approach.

[47] van Merriënboer JJG, Kirschner PA, Frèrejean J (2024). Ten Steps to Complex Learning: A Systematic Approach to Four-Component Instructional Design.

[48] Daniel A, Nagandla K, Daniel S (2025). Application of Gagné’s instructional design in clinical simulation: enhancing learning in obstetric emergency management. Cureus.

[49] Sim WH (2025). Development and evaluation of a multifaceted intervention program for preventing medication administration errors by nurses. J Patient Saf.

[50] Musharyanti L, Haryanti F, Claramita M (2021). Improving nursing students’ medication safety knowledge and skills on using the 4C/ID learning model. J Multidiscip Healthc.

[51] Barari N, RezaeiZadeh M, Khorasani A, Alami F (2022). Designing and validating educational standards for e-teaching in virtual learning environments (VLEs), based on revised Bloom’s taxonomy. Interactive Learning Environments.

[52] Miranda F de, Santos B dos, Kristman VL, Mininel VA (2025). Employing Kirkpatrick’s framework to evaluate nurse training: an integrative review. Rev Latino-Am Enferm.

[53] Sweller J (2020). Cognitive load theory and educational technology. Educ Technol Res Dev.

[54] Lapierre A, Arbour C, Maheu-Cadotte MA, Vinette B, Fontaine G, Lavoie P (2022). Association between clinical simulation design features and novice healthcare professionals’ cognitive load: a systematic review and meta-analysis. Simul Gaming.

[55] Persico L, Ramakrishnan S, Wilson-Keates B (2025). Healthcare simulation standard of best practice prebriefing: preparation and briefing. Clin Simul Nurs.

[56] Cho MK, Kim MY (2024). Enhancing nursing competency through virtual reality simulation among nursing students: a systematic review and meta-analysis. Front Med (Lausanne).

[57] Escobar-Castillejos D, Noguez J, Neri L, Magana A, Benes B (2016). A review of simulators with haptic devices for medical training. J Med Syst.

[58] Lee H (2025). A unified conceptual model of immersive experience in extended reality. Comput Hum Behav Rep.

[59] Davies AJ, Krame G (2024). Measuring the level of fidelity required for transfer of learning in simulation-based learning exercises for novice and experienced practitioners. Simul Gaming.

[60] Lavoie P, Deschênes MF, Nolin R (2020). Beyond technology: a scoping review of features that promote fidelity and authenticity in simulation-based health professional education. Clin Simul Nurs.

[61] Hamstra SJ, Brydges R, Hatala R, Zendejas B, Cook DA (2014). Reconsidering fidelity in simulation-based training. Acad Med.

[62] Andersen MS, Makransky G (2021). The validation and further development of a multidimensional cognitive load scale for virtual environments. J Comput Assist Learn.

[63] Witmer BG, Singer MJ (1998). Measuring presence in virtual environments: a presence questionnaire. Presence.

[64] Hart SG, Staveland LE (1988). Development of NASA-TLX (task load index): results of empirical and theoretical research. Adv Psychol.

[65] Kim YM, Rhiu I (2024). Development of a virtual reality system usability questionnaire (VRSUQ). Appl Ergon.

[66] Foronda CL, Gonzalez L, Meese MM (2024). A comparison of virtual reality to traditional simulation in health professions education: a systematic review. Simul Healthc.

[67] Fransen AF, van de Ven J, Banga FR, Mol BWJ, Oei SG (2020). Multi-professional simulation-based team training in obstetric emergencies for improving patient outcomes and trainees’ performance. Cochrane Database Syst Rev.

[68] Ryan GV, Callaghan S, Rafferty A, Higgins MF, Mangina E, McAuliffe F (2022). Learning outcomes of immersive technologies in health care student education: systematic review of the literature. J Med Internet Res.

[69] Visan IG, Toma CV, Petca R, Petrescu GED, Noditi AR, Petca A (2025). Exploring the efficacy of virtual reality training in obstetric procedures and patient care—a systematic review. Health Care (Don Mills).

[70] Gabr MAM, Sleem WF, El-Wkeel NS (2025). Effect of virtual reality educational program on critical thinking disposition among nursing students in Egypt: a quasi-experimental pretest-posttest design. BMC Nurs.

[71] Chan K, Kor PPK, Liu JYW, Cheung K, Lai T, Kwan RYC (2024). The use of immersive virtual reality training for developing nontechnical skills among nursing students: multimethods study. Asian Pac Isl Nurs J.

[72] Issenberg B, Ostergaard D, Matos F (2026). Setting a research agenda for the use of extended reality in healthcare simulation: an Utstein style meeting. Adv Simul (Lond).

