# Supplementary material for: Integration of Virtual and Augmented Reality Into Obstetric Nursing and Midwifery Education: Systematic Review
Source: JMIR Nurs. 2026 Oct 5;9:e90252. doi: 10.2196/90252 (PMC13639888; doi:10.2196/90252)
Supplement: Supplementary file 1 [file nursing-v9-e90252-s001.pdf]

Integration of Virtual and Augmented Reality into Obstetric Nursing and Midwifery Education: a Systematic Review

Article Types: Review

Search strategy and terms used across databases

Data Base		Query
SCOPUS	TITLE-ABS-KEY	((TITLE-ABS-KEY (nurse* AND midwi*)) OR (TITLE-ABS-KEY (midwi* AND train*)) OR (TITLE-ABS-KEY (midwi* AND student*))) AND ((TITLE-ABS-KEY (simulat*)) OR (TITLE-ABS-KEY ("Educational Technology")) OR (TITLE-ABS-KEY ("3D imaging")) OR (TITLE-ABS-KEY ("Haptic Technology")) OR (TITLE-ABS-KEY (avatar)) OR (TITLE-ABS-KEY ("Three-Dimensional Imaging")) OR (TITLE-ABS-KEY ("augmented reality")) OR (TITLE-ABS-KEY ("High fidelity")) OR (TITLE-ABS-KEY ("virtual reality")) OR (TITLE-ABS-KEY ("extended reality")) OR (TITLE-ABS-KEY ("mixed reality")) OR (TITLE-ABS-KEY (immers*))) AND ((TITLE-ABS-KEY (teaching AND program)) OR (TITLE-ABS-KEY (curriculum)) OR (TITLE-ABS-KEY (training AND programs)) OR (TITLE-ABS-KEY (assess*)) OR (TITLE-ABS-KEY (methodolog* AND approach*)) OR (TITLE-ABS-KEY ("design impact")) OR (TITLE-ABS-KEY (learn* AND concept)) OR (TITLE-ABS-KEY (education* AND impact)) OR (TITLE-ABS-KEY ("instructional design")))
PUBMED	All Fields	((((nurse* midwi*) OR (midwi* train*)) OR (midwi* student*)) AND (((((((((education* impact)) OR (learn* concept)) OR ("instructional design")) OR ("design impact")) OR (methodolog* approach*)) OR (assess*)) OR (Training Programs)) OR (Curriculum)) OR (Teaching Program))) AND (((((((((((immers*) OR ("mixed reality")) OR ("extended reality")) OR ("virtual reality")) OR (simulat*)) OR ("High fidelity")) OR ("augmented reality")) OR ("Educational Technology")) OR ("Three-Dimensional Imaging")) OR ("3D imaging")) OR (avatar)) OR ("Haptic Technology"))
Web Of Science	All Fields	((((nurse* midwi*) OR (midwi* train*)) OR (midwi* student*)) AND (((((((((education* impact)) OR (learn* concept)) OR ("instructional design")) OR ("design impact")) OR (methodolog* approach*)) OR (assess*)) OR (Training Programs)) OR (Curriculum)) OR (Teaching Program))) AND (((((((((((immers*) OR ("mixed reality")) OR ("extended reality")) OR ("virtual reality")) OR (simulat*)) OR ("High fidelity")) OR ("augmented reality")) OR ("Educational Technology")) OR ("Three-Dimensional Imaging")) OR ("3D imaging")) OR (avatar)) OR ("Haptic Technology"))
CINAHL	TX TODO TEXTO	TX (immers* OR mixed reality OR extended reality OR virtual reality OR simulat* OR High fidelity OR augmented reality OR Educational Technology OR Three-Dimensional Imaging OR 3D imaging OR avatar OR Haptic Technology)<br>AND TX (education* impact OR learn* concept OR instructional design OR design impact OR methodolog* approach* OR assess* OR Training Programs OR Curriculum OR Teaching Program)<br>AND TX (nurse* midwi* OR midwi* train* OR midwi* student*)<br>NOT TX (medical students or medicine students or students in medicine or medical education)

Inclusion and exclusion criteria of the systematic review

	Inclusion Criteria	Exclusion Criteria
Population	Undergraduate nursing students,<br>Postgraduate obstetric nursing students,<br>Certified obstetric nurses and midwives (nurse-midwives, midwives),<br>Undergraduate or postgraduate midwifery students	Studies involving multiple health professions that do not separately report or clearly describe data specific to nursing or midwifery students or professionals.
Intervention	Virtual Reality (VR) using head-mounted display (HMD) devices, 3D environments, and first-person interaction.<br>Augmented Reality (AR) with digital overlay on the real world, using AR display devices.	Screen-based technology or gamification without immersion; VR studies that do not allow active first-person interaction; AR studies lacking practical interactivity with digital elements; studies using physical simulations (e.g., mannequins) or non-immersive technologies such as videos or e-learning.
	Educational programs integrating simulation-based scenarios within obstetric care education	Training conducted outside educational contexts (e.g., equipment evaluation); simulations aimed at teaching general nursing care without a specific focus on obstetric care or without scenarios clearly identified as obstetric.
Type of Study	Experimental, quasi-experimental, and observational studies. Only full-text, peer-reviewed articles published in academic journals were included.	Opinion papers, editorials, and case reports; studies that mention VR or AR but do not clearly describe the devices used, the type of interaction, or the evaluated outcomes.
