# Supplementary material for: Integration of Virtual and Augmented Reality Into Obstetric Nursing and Midwifery Education: Systematic Review
Source: JMIR Nurs. 2026 Oct 5;9:e90252. doi: 10.2196/90252 (PMC13639888; doi:10.2196/90252)
Supplement: Supplementary file 3 [file nursing-v9-e90252-s002.docx]

**Table S1**. Assessment of the methodological quality of the experimental studies included in the review, according to the JBI tool.

| Study | Bias Related to  Selection and allocation | | | Bias Related to  Administration of intervention | | | | Bias Related to  Assessment, detection, and measurement of the outcome | | | Bias Related to  Participant retention | Statistical conclusion validity | |
| --- | --- | --- | --- | --- | --- | --- | --- | --- | --- | --- | --- | --- | --- |
|  | True randomization | Allocation concealed | Groups similar at baseline | Participants blinded to treatment | Treatment deliverers blinded to assignments | Treatment groups treated identically other than intervention | Outcome assessors blind to treatment assignment | Outcomes measured in the same way | Outcomes measured in a reliable way | Follow up Complete or group variances described and analysed | Participants analyzed in randomized groups | Appropriate Statistical analysis used | Trial design appropriate, deviations addressed |
| Umoren et al. [27] | Yes | Unclear | Yes | No | Unclear | Yes | No | Yes | Yes | Yes | Yes | Yes | Yes |
| Öner and Turfan [25] | Yes | Unclear | Yes | No | No | Yes | No | Yes | Yes | Yes | No | Yes | Yes |

### **Table S2.** Assessment of the of the methodological quality of the quasi-experimental studies included in the review, according to the JBI tool.

| Study | Bias Related to  Temporal precedence | Bias Related to  Selection and allocation | Bias Related to  Confounding factors | Bias Related to  Administration of intervention/exposure | Bias Related to  Assessment, detection, and measurement of the outcome | | | Bias Related to  Participant retention | Statistical conclusion validity |
| --- | --- | --- | --- | --- | --- | --- | --- | --- | --- |
|  | Clear cause-effect temporal order | Presence of control group | Similar participants across groups | Similar care aside from the intervention | Multiple outcome measurements | Outcomes measured the same way across groups | Outcomes measured reliably | Complete or addressed follow-up | Appropriate statistical analysis |
| Vogel et al. [24] | Yes | No | Yes | Unclear | No | Yes | No | Yes | Yes |
| Zhao et al.  [26] | Yes | Yes | Yes | Yes | No | Yes | No | Yes | Yes |

**Table S3.** Methodological quality of included studies according to MERSQI domains.

|  |  | Umoren et al. [27] | | Öner and Turfan [25] | | Vogel et al. [24] | | Zhao et al. [26] | |  |
| --- | --- | --- | --- | --- | --- | --- | --- | --- | --- | --- |
| Domain | Item | Score | Justification | Score | Justification | Score | Justification | Score | Justification | |
| Study design | 1.Study design | 3 | RCT | 3 | RCT | 1,5 | pretest and posttest | 2 | Nonrandomized, 2 group | |
| Sampling | 2.No. of institutions studied | 1,5 | 20 institutions | 0,5 | 1 institution | 0,5 | 1 institution | 0,5 | 1 institution | |
|  | 3.Response rate % | 1,5 | ≥ 75% | 1,5 | ≥ 75% | 1,5 | ≥ 75% | 1,5 | ≥ 75% | |
| Type of data | 4.Type of data | 3 | Objective | 3 | Objective | 1 | Self-assessment. | 3 | Objective | |
| Validity of evaluation instrument | 5.Content | 1 | Reported | 1 | Reported | 1 | Reported | 1 | Reported | |
|  | 6.Internal structure | 0 | Not reported | 1 | Reported | 0 | Not reported | 1 | Reported | |
|  | 7.Relationships to  other variables | 0 | Not reported | 0 | Not reported | 0 | Not reported | 0 | Not reported | |
| Data analysis | 8.Appropriateness of analysis | 2 | Appropriate | 2 | Appropriate | 2 | Appropriate | 2 | Appropriate for study design | |
|  | 9.Complexity of analysis | 1 | Beyond descriptive analysis | 1 | Beyond  descriptive analysis | 1 | Beyond descriptive analysis | 1 | Beyond descriptive analysis | |
| Outcomes | 10.Outcomes | 1,5 | Knowledge, Skills | 1,5 | Knowledge, Skills | 1,5 | Knowledge, Skills | 1,5 | Knowledge, Skills | |
| Total MERSQI |  | 14,5 |  | 14,5 |  | 10 |  | 13,5 |  | |

**Figure S1.** Mean Medical Education Research Study Quality Instrument (MERSQI) scores by domain.

### References

24. Vogel K, Bernloehr A, Willmeroth T, Blattgerste J, Hellmers C, Bauer NH. Augmented reality simulation-based training for midwifery students and its impact on perceived knowledge, confidence and skills for managing critical incidents. Midwifery. Sep 2024;136:104064. [doi: ] [Medline: 38905862]

25. Öner S, Turfan EÇ. Virtual reality versus low level simulation in newborn care teaching in Turkey. Afr J Reprod Health. Jul 31, 2024;28(7):54-60. [doi: ] [Medline: 39097974]

26. Zhao L, Dai X, Chen S. Effect of the case-based learning method combined with virtual reality simulation technology on midwifery laboratory courses: a quasi-experimental study. Int J Nurs Sci. Jan 2024;11(1):76-82. [doi: ] [Medline: 38352279]

27. Umoren R, Bucher S, Hippe DS, et al. eHBB: a randomised controlled trial of virtual reality or video for neonatal resuscitation refresher training in healthcare workers in resource-scarce settings. BMJ Open. Aug 25, 2021;11(8):e048506. [doi: ] [Medline: 34433598]
